# Clinical Spectrum of Hemolytic Anemia in Loxoscelism: Report of Two Cases Highlighting Variable Severity and Management

**DOI:** 10.1155/crh/9200571

**Published:** 2026-01-05

**Authors:** Anas Al-Sadi, Aakriti Adhikari, Israa Jawarneh, Elrazi Ali, Anuj Shrestha

**Affiliations:** ^1^ Department of Medicine, University of Missouri-Kansas City, Kansas City, Missouri, USA, umkc.edu; ^2^ Department of Medicine, King Abdullah University Hospital, Irbid, Jordan, kauh.jo; ^3^ Department of Hematology and Medical Oncology, University of Louisville, Louisville, Kentucky, USA, louisville.edu; ^4^ Richard & Annette Bloch Cancer Center, Truman Medical Center, Kansas City, Missouri, USA

**Keywords:** complement-mediated hemolysis, corticosteroids, hemolytic anemia, loxoscelism, therapeutic plasma exchange

## Abstract

Loxoscelism can cause local as well as systemic manifestations. Hematologic complications of brown recluse spider venom can be life‐threatening. Here, we present two cases of loxoscelism‐mediated hemolysis that highlight the variable clinical presentations and treatment options available based on severity and pathophysiology of hemolysis.

## 1. Introduction

Loxoscelism, or envenomation by the brown recluse spider, was first linked to necrotic skin lesions in 1957 [1]. *Loxosceles reclusa*, the North American brown recluse spider, is primarily distributed in the south‐central and Midwest regions of the United States, with limited verified presence elsewhere [2]. Despite data that suggest *Loxosceles reclusa*, the North American brown recluse spider, is transported and can be found outside endemic areas, diagnosis of brown recluse spiders outside their areas is very rare [3]. Recluse spiders can be found in areas of high human footprint, such as bedrooms, kitchens, and bathrooms [3, 4], threatening people living in endemic areas.

Here, we present two cases of loxoscelism‐mediated hemolytic anemia. One case involved severe hemolytic anemia requiring therapeutic plasma exchange (TPE), while the other was milder and managed successfully with support from packed red cell transfusion alone. This contrasting severity highlights the clinical variability of loxoscelism‐related hemolytic anemia and the need for individualized management.

## 2. Case 1

**Figure 1:**
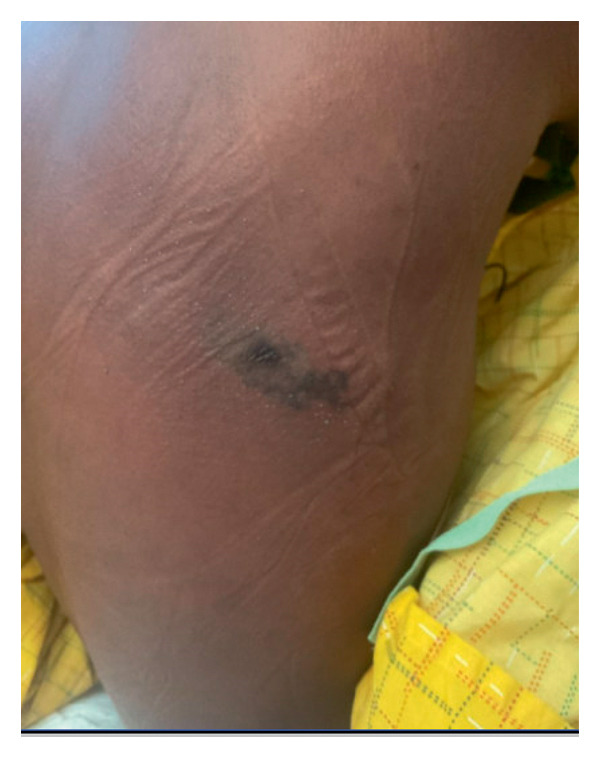
A hyperpigmented, irregularly bordered patch.

The first patient is a 26‐year‐old male patient who presented to the emergency room with progressive generalized weakness for one week. Fatigue was severe enough to make him unable to carry out his daily physical activity. He also reported diffuse arthralgia, chills, and a dry cough. He had reported red urine over the last few days and an itchy, painful skin rash on his back. Two weeks before the presentation, he had a spider bite at the site of the rash. He did not have any significant medical or surgical history. He was not on any regular medications. On initial assessment, the patient looked sick and in distress. He was tachycardic at 110 beats/min and tachypneic at 24 breaths/min, and his temperature was 38 °C. Cardiovascular, respiratory, and gastrointestinal system examinations did not reveal any abnormalities. His skin lesion on the back is shown in Figure 1.

His initial blood work was remarkable for hemoglobin of 11.0 g/dL, white cell count of 17,600/mm^3^, platelet counts of 154,000/mm^3^, international normalized ratio (INR) 1.43, PTT 34 s, fibrinogen level 210 mg/dL, total bilirubin 2.3, direct bilirubin 1.1, and normal liver and kidney functions.

**Figure 2:**
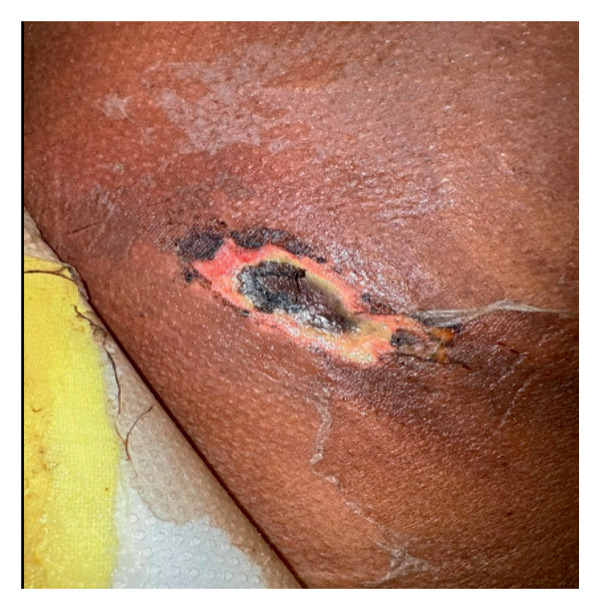
A necrotic skin ulcer with a central black eschar surrounded by an erythematous and inflamed base, with peripheral areas of peeling and dry desquamation.

Over the next 2 days, and despite being treated with broad‐spectrum antibiotics vancomycin and tazobactam‐piperacillin and supportive care with intravenous fluids and antipyretics, he continued to spike high‐grade fever, and his skin lesion became indurated with black necrotic tissue as shown in Figure 2. He became more tachycardic and tachypneic, his lactate was 11.8 mmol/L, and creatinine increased from 1.1 to 1.5 mg/dL. He showed evidence of progressive, severe hemolytic anemia as shown in Table 1.

**Table 1:** Laboratory trends over three days of Patient 1 showing progressive hemolysis, with a sharp decline in hemoglobin, rising bilirubin, hemoglobinuria without red blood cells, elevated LDH, and low haptoglobin.

	Day 1	Day 2	Day 3	Reference ranges
Hemoglobin	11 g/dL	8.3 g/dL	3.6 g/dL	12–16 g/dL
Bilirubin	2.3 mg/dL	5.2 mg/dL	14.3 mg/dL	0.3–1.2 mg/dL
Urine analysis	Trace blood, no RBCs	++ blood, no RBCs		< 5 RBC, negative blood
Lactate dehydrogenase	—	Hemolyzed	1550 U/L	135–250 U/L
Haptoglobin	—	—	6.5 mg/dL	36–195 mg/dL

**Figure 3:**
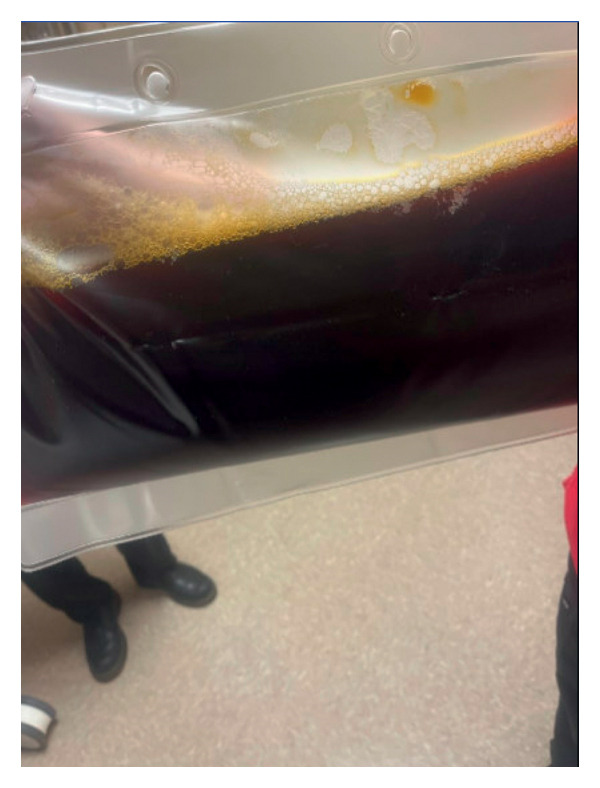
Dark brown to black discoloration of plasma collected during therapeutic plasma exchange, consistent with massive intravascular hemolysis and the presence of free hemoglobin.

His peripheral smear was remarkable for absolute neutrophilia with left myeloid shift, toxic granules, and Dohle bodies. No schistocytes were seen. Direct antiglobulin test (DAT) (Coombs test) was positive for complement 3 (C3) antibodies. Details of immunohematology testing included DAT performed via the column agglutination method without potentiating agents, eluate was not performed, and indirect antiglobulin test was negative. His other workup–autoimmune panel, HIV, and blood cultures were all unremarkable. Given his clinical and biochemical deterioration, he was shifted to the critical care unit. He was diagnosed with loxoscelism with possible complement‐mediated hemolytic anemia. Because of the critically low value of hemoglobin 3.6 g/dL before the first TPE, he was given a stress dose of methyl prednisone for five days and he was transfused with 2 units of packed red blood cells (RBCs) right before the procedure, which brought the level to a safer range 5.8 g/dL. The exchange itself was carried out using albumin as the replacement fluid to help maintain the volume status. The goal of TPE in this case was to stabilize the hemoglobin by removing venom components and complement‐activating factors and to prevent further rapid drops. Two sessions of TPE were performed with 1:1 volume exchange using albumin, replacing approximately one plasma volume per session. LDH was reported as “hemolyzed” on Day 1 and 1550 U/L on Day 3. Reticulocyte and haptoglobin data were limited. Lactate elevation lacked a clear etiology (shock vs. sampling vs. tissue hypoxia). Sepsis was considered but adjudicated against venom‐mediated hemolysis. His plasma is shown in Figure 3. Hemoglobin stabilized and improved without further transfusions or TPE.

## 3. Case 2

The second patient is a 23‐year‐old female patient who presented to the emergency department (ED) for the first time after seeing a brown spider, which she suspected had bitten her. She reported tingling pain and mild skin discoloration in that area.

**Figure 4:**
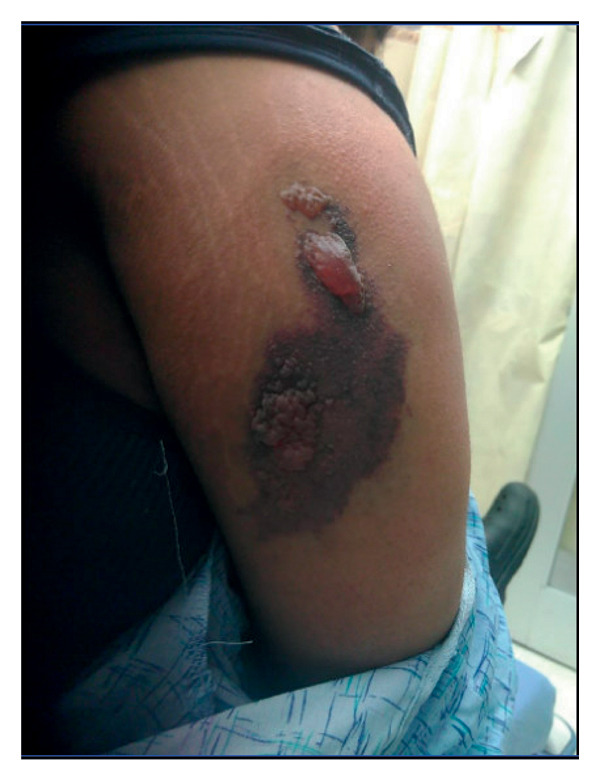
A dark, irregular lesion on the upper arm with crusting, erosions, and flaccid bullae.

Her blood work revealed hemoglobin level 10.1 g/dL, WBC 16,000/mm^3^, and INR 1.09. Her kidney function test, liver function test, and bilirubin were all within normal range. She was discharged home on oral doxycycline. Two days later, she presented again to the ED with worsening pain and skin lesion at the site of the bite as shown in Figure 4, associated with low‐grade fever documented in the ED as 37.8°C, chills, and palpitations. Then, bilirubin was 1.9 mg/dL and INR was 1.54. Hemoglobin level, platelet counts, and kidney function tests were all at baseline. She was recommended for admission, but she preferred to go home and return if she did not improve.

**Figure 5:**
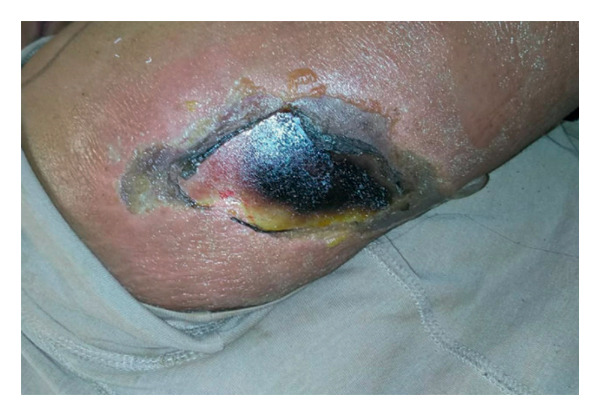
A necrotic ulcer on the lateral thigh with a central area of black eschar, surrounded by yellow slough and erythematous, moist skin.

Five days after the spider bite, she returned to the ED with worsening local symptoms, fever, and a draining blister at the site. Vital signs were remarkable for temperature 37.8 °C, pulse rate 125 beats/minutes, BP 120/57 mmHg, and oxygen saturation 100% on room air. Physical examination was unremarkable except for an ulcerated lesion at the bite site, as shown in Figure 5.

Significant blood work is shown in Table 2.

**Table 2:** Significant blood work of Patient 2.

	Day 1	Day 2	Day 3	Reference ranges
WBC	25,000/mm^3^	31,000/mm^3^	15,000/mm^3^	4500–11,000/mm^3^
Hemoglobin	7.8 g/dL	6.0 g/dL	7.3 g/dL	12–16 g/dL
Bilirubin	4.3 mg/dL	6.6 mg/dL	2.8 mg/dL	0.3–1.2 mg/dL
Indirect bilirubin	3.2 mg/dL	4.5 mg/dL	1.7 mg/dL	< 0.7 mg/dL
CRP	193 mg/L	—	—	< 10 mg/dL
Lactate dehydrogenase	215 U/L	295 U/L	264 U/L	135–250 U/L
Haptoglobin	55 mg/dL	15 mg/dL	—	36–195 mg/dL
D‐dimer	1527 ng/mL	—	—	< 500 ng/mL
INR	1.37	1.25	1.1	
Corrected Retic. %	3.5%			0.2%–2%

Kidney function test and liver function test remained normal. Her peripheral blood smear revealed neutrophilia and normocytic anemia with no schistocytes. DAT (Coombs test) was negative. The patient required a total of 4 units of PRBCs transfusion to maintain a hemoglobin level > 7.0, and she was treated with intravenous antibiotics for a total of 3 days. The infectious disease team was consulted at Day 3, and they advised to stop the antibiotics. The patient’s condition stabilized with no recurrence of fever, and the skin lesion had improved. Her hemoglobin remained stable without further red cell transfusion.

## 4. Discussion

Brown recluse spider bites can cause significant morbidity related to both local reactions and life‐threatening systemic illnesses. In a single case series, 4 out of 9 patients were admitted to the intensive care units (ICUs) for dermatologic abscess, severe sepsis, and hemolytic anemia [5]. The initial bite is relatively painless, and over hours to a few days, patients develop soreness, pruritus, and dark necrotic lesions, resulting from severe vasoconstriction [6]. This pattern happened in both reported patients. Progressive necrosis results in eschar formation, and a change in color from erythema to violaceous is an important clue to differentiate necrotic from non‐necrotic venomous bites [7]. No relation between the location of the bite and the severity of local reaction or systemic illness was identified [5].

*Loxosceles* venom contains several enzymes, most notably sphingomyelinase D (SMase D), which initiates a cascade of local and systemic effects. This toxin causes direct endothelial damage, activates the complement system, and promotes the release of inflammatory cytokines. In susceptible individuals, this immune activation culminates in complement‐mediated hemolysis, hemoglobinuria and renal impairment [8, 9]. In our first patient, a DAT positive for C3 alone and undetectable haptoglobin suggested activation of the alternative complement pathway, most likely consistent with SMase D–mediated erythrocyte destruction. In contrast, the second patient had a negative DAT despite clinical and biochemical signs of hemolysis, which may suggest venom‐induced red cell fragility or direct lysis independent of immune complex formation [8, 10]. One hypothesis that may explain the exact mechanism is a low level of glycophorin A, as evidenced by a single in vitro analysis of RBCs of 17 patients with brown recluse spider bites [11]. Depletion of glycophorin A could be explained by venom‐induced production of antibody or direct venom effect on the RBC membrane.

We presented two cases of presumed brown recluse spider bites complicated by necrotic skin lesions and acute hemolysis. Differential diagnoses include bacterial necrotic cellulitis, disseminated intravascular coagulation (DIC), and vasculitis. Blood and wound cultures did not grow any organisms, and the peripheral smears did not show evidence of microangiopathic hemolytic anemia to suggest DIC. Given the acute presentation, lack of other systemic signs suggestive of autoimmune disease and the high prevalence of brown recluse spider in Midwest, the most likely diagnosis was systemic manifestation of loxoscelism.

These two cases highlight the diverse clinical presentations of systemic loxoscelism from fulminant complement‐mediated hemolysis to a delayed DAT‐negative lysis of red cells. These two cases highlight several important clinical insights. First, systemic loxoscelism can occur even in adults and should not be overlooked based solely on age or initial appearance. Second, a necrotic lesion in conjunction with early signs of hemolysis or systemic symptoms should prompt urgent evaluation and monitoring. Clinical trajectory, rather than serologic findings alone, should guide decisions regarding aggressive interventions like TPE. A positive DAT for C3 may support complement involvement, but its absence does not exclude the diagnosis [12, 13]. In endemic regions, or when a necrotic skin lesion is present, systemic loxoscelism should remain on the differential for unexplained hemolytic anemia. Third, the diagnosis remains largely clinical, especially in regions where spider identification is uncommon or unavailable, and clinicians should maintain a high index of suspicion when patients present with rapidly evolving skin lesions and unexplained hemolytic anemia. Fourth, in cases of severe hemolytic anemia, it can be managed in the ICU with transfusions, high‐dose corticosteroids, and TPE.

High‐dose corticosteroids have been reported as a treatment of loxoscelism‐mediated hemolytic anemia, especially IgG‐mediated and delayed hemolysis [3, 14].

Although not routinely done, TPE has been used in fulminant systemic loxoscelism to mitigate complement‐mediated hemolysis and venom‐related inflammation [10, 15]. Harry S et al. reported a similar successful outcome using TPE in a patient with refractory loxoscelism‐associated hemolytic anemia [10]. Another successful case by Abraham et al. was in a 17‐year‐old female with systemic loxoscelism following TPE, suggesting it may be a potential treatment for systemic loxoscelism with refractory hemolytic anemia by removing venom‐activated complement components [16]. It is clear that TPE has been attempted in refractory cases to remove circulating venom and/or activated complement; however, evidence is limited to case reports, and risks/benefits should be individualized.

Finally, this case series highlights the importance of increasing awareness among physicians practicing in endemic areas for early recognition of the disease. It also highlights the need for systematic review and meta‐analysis of available data to establish clear guidelines for treating physicians.

**Nomenclature**
°C: Degrees celsius
INR: International normalized ratio
PTT: Partial thromboplastin time
C3: Complement 3
RBCs: Red blood cells
ED: Emergency department
DAT: Direct antiglobulin test
SMase D: Sphingomyelinase D
TPE: Therapeutic plasma exchange

## Consent

The patients had provided informed consent for publication of the cases.

## Conflicts of Interest

The authors declare no conflicts of interest.

## Funding

No funding was received for this manuscript.

## Data Availability

Data sharing is not applicable to this article as no datasets were generated or analyzed during the current study.
